# Phosphorous-Based, Halogen-Free Flame Retardants for Thin, Flexible Polyurethane Artificial Leathers

**DOI:** 10.3390/polym17070841

**Published:** 2025-03-21

**Authors:** Miriam Bader, Maren Lehmann, Michael Meyer

**Affiliations:** FILK Freiberg Institute gGmbH, Meißner Ring 1-5, 09599 Freiberg, Germany

**Keywords:** polyurethane, halogen-free, flame retardant, artificial leather, bentonite

## Abstract

Polyurethane (PUR)-based artificial leathers are often used as interior materials in public area, making flame retardants (FRs) necessary. The mode of action of different FRs varies depending on the chemical class and the structure of the supplied material. Usually, FRs are designed for bulk materials like foams, e.g., for upholstery, the main application of PUR. However, in thin materials, FRs act differently, thus leaving the PUR without sufficient flame resistance. In this study, PUR films and artificial leathers were equipped with twelve commercially available, halogen-free FRs in various concentrations and combinations. Fire resistance was tested with LOI measurements, cone calorimetry, horizontal burning behavior, and thermogravimetric analyses. An organophosphorus FR proved to be the most suited for flame-resistant artificial leather. The LOI was increased from 20 to 24.2%, the peak heat release rate was reduced by about 30%, and the sample was self-extinguishing in horizontal burning behavior. Phosphinates and aluminum trihydroxide were the least efficient FRs. Combinations of bentonite with phosphorus-based FRs showed synergistic effects in reducing the probability of igniting the material. The results demonstrate that sufficient flame retardancy for PUR-based thin materials can be achieved with commercially available halogen-free FRs, paving the way for more sustainable and greener materials by substituting ecologically harmful and health-damaging FRs.

## 1. Introduction

The versatility of the polymer polyurethane (PUR) becomes apparent in its use in rigid or flexible foams, varnishes, or compression molding to mention a few examples [1,2,3]. Another essential application is as a coating material for textiles, with the aim of generating composites with tailor-made properties. These flexible, thin composite materials are primarily applied in technical textiles such as flooring, roof sheeting, and conveyor belts. PUR is also used in coated textiles for clothes and artificial leather. It is important to note that the usage of terms describing synthetic leather-like materials is restricted in the European standard EN 15987 [4]. In this study, the term “artificial leather” is employed to distinguish our material from other coated textiles, whose application range is even greater.

The extensive utilization of artificial leather in various applications, including the interiors of cars, trains, and cruisers, the upholstery of furniture, patient benches in hospitals, and in clothing, shoes, and travel accessories illustrates its significance. Each of these intended purposes requires special material specifications, which are realized by using the relevant PUR base and additives. Conventional PUR is flammable, making flame retardants (FRs) mandatory for many applications [5,6].

Little is known about how different FRs act in materials like artificial leather; the focus is mostly on PUR foams since this is the most common application of PUR [6]. Nevertheless, especially for developing new formulations for artificial leathers, it is essential to know suitable FRs and their concentration ranges. To strengthen sustainability aspects, saving time, energy, and resources are the most impactful factors. The potential to reduce the testing efforts while obtaining comprehensive results was evaluated by focusing on investigations of the functional intermediate layer. Therefore, an extensive comparison of films, equal to the intermediate layer, and the whole product artificial leather was performed. Furthermore, the balance between the minimal necessary amount of FR to obtain a good fire retardancy and the impact on mechanical properties was evaluated. Often, high amounts of FR have negative effects like reduced flexibility and increased brittleness.

**Figure 1:**
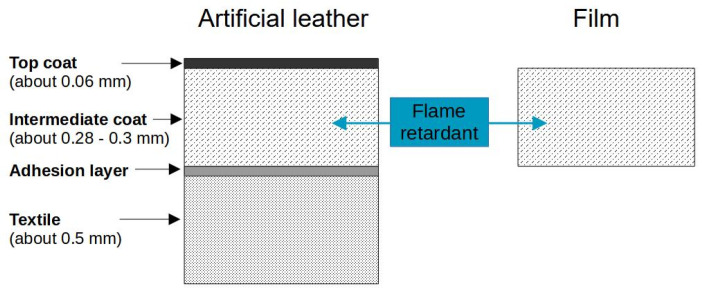
Comparison of the construction of the multi-layer design of artificial leather (**left**) and a single film (**right**).

Flame retardants act, basically, through three mechanisms: radical scavenging, char formation, and intumescence. Halogenated FRs are effective and inexpensive [7]. They show a high flame retardant efficiency by acting in the gas phase as radical scavengers. In the case of fire, the fire itself may be suppressed by the halogenated FRs, but it is accompanied by an intense smoke formation. Toxic and corrosive halogen-containing decomposition products are released [6,8,9]. The consequences are high material damage and toxic effects on humans and the environment. Therefore, the use of halogen-containing substances is under discussion due to negative aspects like persistence, enrichment in the environment, and hormone-like activities. The search for alternatives already started ten years ago [10,11,12]. Today, regulatory restrictions are making halogen-free alternatives necessary. In contrast, phosphorous compounds are promising alternatives for replacing common halogen-containing molecules. Phosphorous flame retardants are diverse, as are their modes of action. Some are active in the condensed phase and some in the gas phase, depending on the chemical environment and oxidation state of phosphorus. Phosphinates are active in the gas phase by stopping the radical mechanism of the combustion process. Char formation and intumescence are the mechanisms of the condensed phase. The former is induced by the dehydration of the polymeric structure followed by the generation of double bonds, cyclization, and crosslinking, resulting in forming a carbonaceous layer. This char shield reduces the release of volatile, oxidizing compounds and serving as a protective layer. Organic phosphates and phosphate esters belong to the char-forming FRs. For intumescence, an additional blowing component is needed, in most cases an N-containing substance [13,14,15,16,17,18,19]. Ammonium polyphosphate (APP) is a typical example. Using a combination of phosphorus and nitrogen functionalities often leads to synergistic effects [13,20,21]. Various versions of P/N FRs are commercially available, but most of them were developed for bulk materials, especially for soft and rigid polyurethane foams, respectively. Only a few investigations are available about FRs for thin-film polymer composites. However, it is necessary to provide artificial leathers with the best flame retardancy possible for use in public areas or transport, such as airplanes, ships, or trains, with no option of escape. Selected studies investigated the flammability of artificial leather in general [22] or for the combination of individual flame retardants and one material [6,23,24]. For instance, Zhao et al. incorporated only 4% of APP in a reactive polyurethane with an enhancing effect on flame retardancy for an artificial leather based on PUR [23]. Furthermore, biobased compounds like lignin are becoming increasingly important. Modified with phosphor and used as a FR, the flame retardant effects are detectable [24,25], but these biobased FRs are not commercially available. In our study, we used twelve commercially available additive FRs: eleven phosphorous FRs and aluminum trihydroxide (ATH). According to their technical data sheets, they were classified into different chemical compound classes: phosphinates (3), organic phosphorus (1), phosphates (2), ammonium polyphosphates, partly with melamine (5), and aluminum trihydroxide (1). All FRs were incorporated in a film and in the intermediate layer of an artificial leather manufactured from an aromatic polyurethane high-solid prepolymer (Figure 1).

The influence of varying FR concentrations was examined, as well as different FR combinations. To verify the transferability to other PUR systems, a second aromatic and an aliphatic high-solid PUR prepolymer were included in a test series. Additionally, two different synergists, a clay [26,27,28,29] and a hindered amine light stabilizer (HALS) [17,30] were tested in combination with selected FRs to enhance flame retardancy. Clays are low-cost additives with ubiquitous occurrence, which improve gas barrier properties. The clays consist of layered silicate sheets and exhibit a labyrinth effect which may hinder and delay heat and mass transfer in a burning material [27,28,31]. Horizontal burning behavior, thermogravimetric analyses, limiting oxygen index (LOI), and cone calorimetry (CC) were used to characterize the burning and decomposition behavior. To the authors’ knowledge, there is currently no study that considers such a broad range of different FRs on PUR-based artificial leather with this intensity.

## 2. Materials and Methods

### 2.1. Materials

Two aromatic high-solid PUR prepolymers and the corresponding crosslinker were provided by Stahl Holding BV and Covestro AG, respectively. The aliphatic high-solid PUR prepolymer with the corresponding crosslinker was supplied by Stahl Holding BV. These prepolymers were applied in the intermediate layer of the composite. The topcoat and adhesive layer consisted of waterborne polycarbonate ester PUR dispersions (Covestro AG, Leverkusen, Germany). The twelve FRs utilized in this study were supplied by Clariant, Huerth, Germany (aluminum-diethyl phosphinate, phosphinate, APP, and melamine-coated APP); Thor GmbH, Speyer, Germany (phosphinate and organic phosphorus compound); Lanxess, Leverkusen, Germany (phosphates); Chemische Fabrik Budenheim KG, Budenheim, Germany (melamine- or silane-coated APP and APP intumescence mixture) and Sigma Aldrich, Darmstadt, Germany (ATH). The phosphorous FRs exhibited phosphorus contents ranging from 8.5% to 32%. Their properties are summarized in Table 1. Five of these FRs were selected for extended studies; they are highlighted in gray. The clay bentonite (Euro OTC Pharma GmbH, Bönen, Germany) and the N-alkoxy hindered amine light stabilizer (BASF Schweiz AG, Kaisten, Switzerland) were used as synergistic additives.

### 2.2. Preparation of Films and Artificial Leathers

The artificial leathers were manufactured in a transfer coating process with a lab coater (Labor-Coater Mathis LTE-SM). The composition and preparation conditions are summarized in Table 2.

The samples of artificial leather (Figure 1) were prepared by first applying the topcoat consisting of 100 phr waterborne PUR, 1 phr thickening agent, and 0.5 phr expiration aids on a transfer paper. Next, the intermediate layer consists of the aromatic or aliphatic high-solid prepolymer, respectively. This polymer compound, the corresponding crosslinker, and an FR (20 phr, if not otherwise stated) were mixed and coated onto the topcoat. This study was performed with HS 1 as the polymer. To prove the transferability of the results, HS 2 and HS 3 were used for selected additional setups. Non-FR synergists were added to the intermediate layer of the artificial leather, which also contained 20 phr of phosphorous FR. The HALS component was incorporated in a concentration of 1 phr as recommended by the supplier. The clay bentonite had a concentration of 2 phr in all formulations except for the ATH-containing one. Due to the high viscosity, the processing of the formulation with ATH and bentonite was not possible.

Subsequently, the adhesion layer (100 phr waterborne PUR, 6 phr crosslinker, 1 phr thickening agent, and 0.5 phr expiration aids) was applied to the intermediate layer and laminated with cotton (area weight 115 g m^−2^). When preparing the freestanding films, the high-solid prepolymer mixture was directly applied onto transfer paper and cured, similar to the intermediate layer of the composite.

The concentration of the FR either in film or in the intermediate layer was 20 phr. An exception was made for studies on concentration dependence, where amounts between 5 and 30 phr of the corresponding FR were used.

All samples were manufactured as laboratory samples with a size of 240 mm × 240 mm.

### 2.3. Characterization Methods

#### 2.3.1. Cone Calorimetry

Cone calorimetry measurements were performed with a cone calorimeter manufactured by Wazau (Dr. Ing. Georg Wazau Mess- + Prüfsysteme GmbH, Berlin, Germany) with a distance of 60 mm between the sample and the cone-shaped heater with a heat flux of 25 kW m^−2^. Following ISO 5660-1 [32], samples with a size of 100 mm × 100 mm were investigated in triplicate. Deviating from the standard, which requires a sample thickness of 6 mm, the thickness of investigated samples was about 0.8 mm. TTI (time to ignition), PHRR (peak heat release rate), THR (total heat release), and TSP (total smoke production) were the obtained values, which were used for the evaluation and a comparison of different samples.

#### 2.3.2. Limiting Oxygen Index

The limiting oxygen index (LOI; NETZSCH, TAURUS Instruments AG, Weimar, Germany) measurements were performed in accordance with the standard DIN EN ISO 4589-2 [33], employing specimen form V (sample size—120 mm × 50 mm). The samples were clamped in a vertical holding frame and exposed to a 16 mm diameter flame for five seconds. If the sample did not ignite, the procedure was repeated after five seconds for a total of 30 s. The oxygen concentrations were varied as defined in the standard. Information about the inflammability of a material was obtained. The values represent at which oxygen concentration the sample can be ignited and the sample burns. If the LOI is below 20.95%, the material is flammable; if it is between 21% and 28%, the material burns slowly, and if it is above 28%, the material is self-extinguishing [34].

#### 2.3.3. Horizontal Burning Behavior

The burning rate was determined in a small-scale burning test in accordance with DIN 75200 [35]. Specimens of 120 mm × 75 mm × ~0.8 mm were exposed to a small, 38 mm flame for 15 s. Information about the burning behavior was obtained. If the samples began to burn, the burning rate was calculated and expressed in mm/min. Burning behavior tests were carried out in triplicates. The threshold of burning rate is 100 mm/min for some standards (like FMVSS 302 and TL1010).

#### 2.3.4. Thermogravimetric Analysis

The combustion behavior of films and artificial leathers was analyzed by thermogravimetric analysis (TGA) using the TGA/DSC 3+ (Mettler-Toledo GmbH, Giessen, Germany) with a ceramic sensor (TGA DSC Sensor LF, Mettler-Toledo GmbH, Giessen, Germany). A linear heating rate of 10 K min^−1^ was applied in an atmosphere of synthetic air. The weight of all investigated artificial leathers was between 6.5 and 7.5 mg, the weight of all investigated films was between 3.5 and 5.5 mg, and the weight of the investigated FR was about 7 mg. Samples in an open Al pan were examined under an airflow rate of 20 mL min^−1^ at a temperature ranging from 25 to 600 °C. The decomposition temperature at 10% weight loss, reversal point of the sigmoidal curve, and residual mass were determined. The first derivative of the relative mass concerning the temperature was formed and multiplied by −1 for better detection of the reversal points. TGA is a prevalent method for studying the mechanism and kinetic decomposition of materials, providing insights into their thermostability and the effectiveness of FRs [36]. Since it is a fast method with low material consumption, it was chosen for comparison of all FRs and for proof of whether all FRs in one chemical group have the same effect on flame retardancy.

#### 2.3.5. Mechanical Characterization

Tensile strength and elongation at break were performed according to standard DIN EN ISO 527-3:2019 [37]. The flex-resistance test was conducted in accordance with the standard ISO 32100:2010 [38].

## 3. Results and Discussion

### 3.1. Flame Retardant Comparison

Given the diversity of FR mechanisms of chemical compound classes, a comparative study was performed to assess the potential for reducing the screening of materials. Therefore, TGA and LOI measurements were performed.

The analysis of decomposition curves and residual masses by TGA has been shown to provide insights into the mechanisms of FRs. The residual masses are indicators for char yields. Consequently, the higher the residual mass, the higher the amount of char. In the case of FR activity being confined to the condensed phase, the effectiveness of flame retardancy is also higher [20,39].

**Figure 2:**
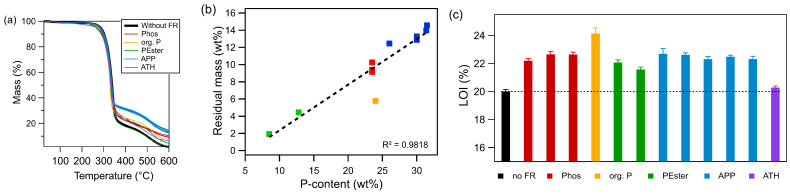
(**a**) Thermogravimetric results of artificial leathers with different flame retardants, (**b**) correlation of residual masses after thermogravimetric analysis and phosphorus content of FRs (org. P value is not included in regression determinant), and (**c**) limiting oxygen index values (HS 1, [FR] = 20 phr). (Abbreviation: Phos—phosphinates; org. P—organic phosphorus compound; PEster—phosphate ester; APP—ammonium polyphosphate; ATH—aluminum trihydroxide).

The decomposition curves of artificial leathers (Figure 2a) with different FRs illustrate the mass loss as a function of temperature. It was observed that all artificial leathers exhibited comparable decomposition temperatures, ranging from 324 to 337 °C, irrespective of the type of FR employed. However, the residual masses of the FRs varied according to the FR chemical class. Samples with APP-based FRs had the largest residual mass (13.4 ± 0.8%) followed by the phosphinate-based FR (9.6 ± 0.5%). Notably, all other samples had residual masses that were either similar to or slightly larger than the FR-free artificial leather. A linear relationship was observed between residual mass and P content, with a regression coefficient of 0.9818 if the result of the artificial leather with org. P was excluded. Compared to the supplier-given P content of org. P, the measured residual mass of 5.9 ± 0.1% was too low (see yellow square in Figure 2b). This observation suggests the potential for the formation of volatile P-containing substances and their subsequent action in the gas phase. Results obtained from the other FRs did not show any indication of a similar behavior. The direct proportionality between increasing residual masses and P content indicate the formation of a char layer which increases with rising P content. The char layer serves as a protective barrier on the surface of the polymer. Consequently, the heat and mass transfer to the combustible material appear to be suppressed [20,40]. However, not only the phosphorus concentration of FR but also its mechanism of action strongly influences the material’s flame retardancy. The sample with org. P showed the highest LOI value (Figure 2c), despite not having the highest P content. The formation of a char layer, along with the generation of volatile compounds, contributed to the observed highest FR efficiency.

All artificial leather samples with FRs showed an increased LOI in comparison to the reference without FR, indicating that FRs effectively reduced the inflammability (Figure 2c). Furthermore, FRs of the same chemical class showed similar results. All phosphinate-containing materials (red) had similar LOIs with a variance between samples of 0.3%. The APP-containing samples (blue) showed a comparable LOI (variance 0.3%) as Phos-containing artificial leathers. PEster were the only FRs which caused a higher variance between the two samples. One of the Pester FRs was an oligomeric compound, while the other was a triphenyl phosphate. The artificial leather containing the oligomeric PEster showed a higher LOI compared to the one containing the triphenyl phosphate. Since compatibility with the polymer and steric effects influence the burning behavior, it is deduced that the oligomeric PEster would exhibit enhanced compatibility with high-solid PUR compared to the triphenyl phosphate [15]. Additionally, the P content of the latter was over 4% lower, which is a significant amount of P, particularly in such thin materials.

The other two FRs, APP and Phos, showed similar results within their respective chemical functionality group but could be distinguished from other FR classes. Therefore, only one representative member of each chemical FR group was included in the following experiments.

### 3.2. Comparing Results of Films and Artificial Leathers

Films with the same composition as the intermediate layer of the artificial leathers were compared to artificial leathers regarding flame retardant effects and decomposition behavior. The FR concentration was always 20 phr.

**Figure 3:**
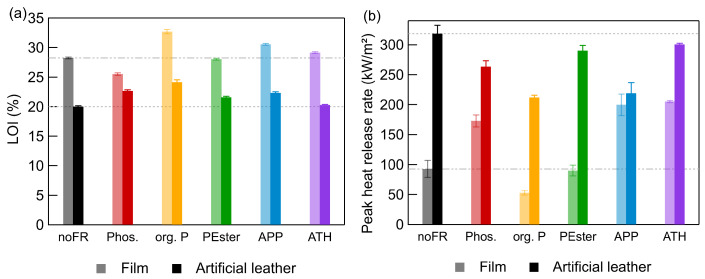
Comparison of characteristic burning behavior values of films (lighter color) and artificial leathers (darker color) with 20 phr FR: (**a**) LOI and (**b**) peak heat release rate (cone calorimetry). Dotted horizontal lines indicate the values of reference materials without FR. (Abbreviation: Phos—phosphinates; org. P—organic phosphorus compound; PEster—phosphate ester; APP—ammonium polyphosphate; ATH—aluminum trihydroxide).

The reference artificial leather without any FR had an LOI of 20.0 ± 0.2% (Figure 3a) and was, therefore, not self-extinguishing under standard conditions. All artificial leathers with a FR exhibited a higher LOI. In contrast to artificial leather, the reference film had a LOI of 28.2 ± 0.2% and was, therefore, self-extinguishing, whereas most films with FR showed a lower or similar LOI (Figure 3a). Exceptions were the films with org. P and APP, which had higher LOIs of 32.7 ± 0.4% and 30.6 ± 0.2%, respectively. A notable observation was the occurrence of an afterglow in all artificial leathers containing phosphorous FR upon burning.

ATH showed no effect in films nor in artificial leather. As demonstrated by Weil et al., Thirumal et al., and Duquesne et al., for polymers in general, as well as for PUR foams, high loadings (up to 50–60%) of ATH are necessary to achieve sufficient flame retardancy [41,42,43]. The incorporation of such high concentrations of solid additives into the formulation of films and artificial leathers will induce significant challenges during the manufacturing process. Moreover, even if these materials could be produced, their mechanical properties, being thin and flexible, would be greatly reduced. Therefore, 60% of ATH is not feasible.

According to ISO 5660-1 [32] for CC measurements, materials should have a thickness that exceeds 6 mm. Depending on the heat release curves, different burning mechanisms can be derived. However, the artificial leather samples based on high solids had a thickness of approximately 0.8 mm. The heat release curve of these samples resulted in the formation of sharp peaks. Schartel et al. discussed the thermal analysis of such so-called thermally thin samples. Their findings indicated that no flame retardant mechanism can be derived from the sharp peak of heat release. The reason is that the sample undergoes complete combustion instantaneously following ignition [44,45]. Nevertheless, we applied CC for our artificial leathers since the thickness of all tested materials was the same, and, therefore, the obtained values were comparable with one another. The PHRRs of the artificial leathers were larger than the PHRRs values of films (Figure 3b). The higher content of flammable materials in artificial leathers relative to films of the same area resulted in increased heat release. The cotton as a textile carrier on its own already had a PHRR of 68 ± 13 kW m^−2^. The adhesive and topcoat contributed additionally.

In most cases, the PHRR was higher in films with FRs than without FRs. Only films with org. P showed a decreased PHRR, hinting to their improved flame retardancy. Photographs of the burned films are presented in Figure S1. A char formation of the film with org. P is visible. The film containing APP showed a similar char/intumescence formation but a higher PHRR than the reference film. Indicating that the char formation is not the only active mechanism in flame retardancy for org. P. These findings support the results of TGA. All artificial leathers supplied with FRs showed lower PHRRs than their FR-free reference material. Consequently, the incorporation of 20 phr of a phosphorous FR in the intermediate coat of artificial leathers resulted in a discernible enhancement in fire retardancy. However, when ATH was used, no effect was observed. The results of the films differed significantly from one another. The films with the FR org. P demonstrated positive fire-retardant effects in the LOI and CC, while films with APP or ATH had contradictory results. The presence of PEster in films appeared to have no effect, as evidenced by the LOI and CC results. In conclusion, there is no transferability from the results of films to those of artificial leathers. The underlying mechanisms of heat and mass transfer in composite materials differ significantly from those of self-supporting films.

**Figure 4:**
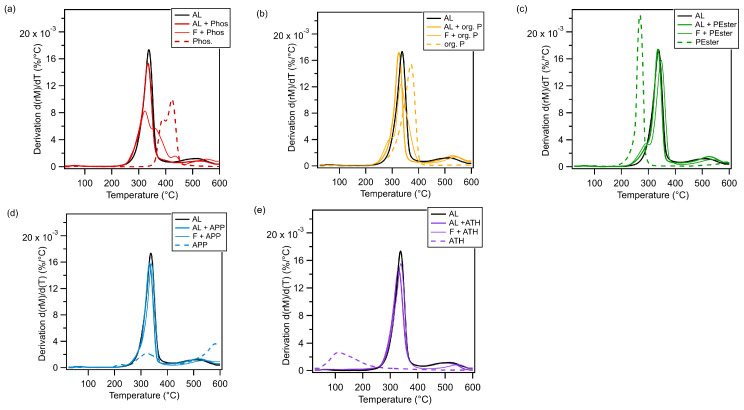
Thermograms of pure FR, artificial leather (AL) with and without FR, and films (F) with FR (**a**) phosphinates (Phos), (**b**) an organic phosphorus compound (org. P), (**c**) an organic phosphorus compound (PEster), (**d**) ammonium polyphosphate (APP), and (**e**) aluminum trihydroxide (ATH).

A comparative analysis was conducted using TGA measurements (Figure 4) to assess the decomposition of pure FR, artificial leathers with and without FR, and films with FR. Phos was the only FR that decomposed at significantly higher temperatures compared to the artificial leathers and films, without FR or with the other FRs. Org. P and APP showed decomposition temperatures which were similar to that of FR-free artificial leather. For PEster and ATH, lower temperatures were observed. The decomposition of the artificial leathers with FR followed a one-step reaction, as evidenced by the presence of a single maximum in the derivatives, resulting in a behavior analogous to that of the reference material. No consistent decomposition behavior could be observed for the films. Especially, films with Phos showed a multistage decomposition process with two maxima at temperatures higher than the decomposition temperature of the reference material. Films with org. P and PEster had a minor shoulder at lower temperatures compared to the decomposition of the film without FR. The residual mass was found to be the lowest for the film and artificial leather without any FR, while the highest was determined for the materials with APP. Generally, the artificial leathers had a lower residual mass than the films since the amount of FR per mass is lower than the one for films.

The results of the three methods, LOI, CC, and TGA, for both types of materials are summarized in Table 3. The higher mass of flammable material per test sample of artificial leather compared to the films resulted in a lower ignition resistance (lower LOI) and a higher heat release upon ignition (higher PHRR). Based on weight, 20 phr FR in films would equal approximal 30 phr FR in artificial leather. But only increasing the FR did not lead to an improvement. For instance, the LOI of the film with 20 phr org. P was 32.7 ± 0.4%, while the corresponding artificial leather with 30 phr org. P exhibited an LOI of 25.1 ± 0.1%.

Therefore, the crucial factor is not necessarily the FR concentration but rather the FR’s functionality, the polymer material, and its configuration (i.e., one layer or composite). The presence of Phos in films has been shown to result in an enhancement of flammability; conversely, in the case of artificial leather, a reduction in flammability has been observed. Despite the similarity in composition between the film and the intermediate layer, it is the covering of the layer with a topcoat and the textile that proves decisive. Presumably, this coating provided the FR in the intermediate layer with sufficient time to become active. The inefficiency of Phos in PUR films is further substantiated by the findings of thermogravimetric analyses. The decomposition process in this case was multistage, with stages occurring at temperatures higher than those observed in artificial leather (Figure 4a). This indicates the release of scavenging radicals from Phos, which are known to be active in the gas phase [15,43,46]. If the FR-equipped intermediate layer was covered from one side with the topcoat and from the other with a textile, the material decomposed in a single step, and a fire-retardant action was detected.

Comparing the test results for films and artificial leathers with the same FR concentration reveals two key observations. Firstly, the flame retardancy of films can either be improved or worsened depending on the method used. This suggests that while FRs in films may enhance inflammability, they may not necessarily have a positive effect on burning behavior. For instance, the use of APP results in an increased LOI, leading to a higher flame resistance, but the PHRR almost doubled. While the ignition of APP-containing materials is less likely to occur, the release of heat upon combustion is greater. In contrast, the trend observed for artificial leathers is consistent across all methods. The incorporation of FRs into artificial leathers has been shown to enhance flame retardancy, as evidenced by an increase in the LOI, a decrease in the PHRR, and a reduction in burning rate.

Secondly, the findings indicate that the results of films cannot be extrapolated to artificial leathers. A flame retardant effect has been observed in artificial leathers with Phos but not in films with equivalent flame retardant properties. In the case of org. P as an FR, films and artificial leathers showed satisfactory FR properties. However, it should be noted that not all FRs showed the same efficacy in both films and artificial leathers, which hinders the establishment of a universal prediction model. To ensure the efficacy of any given FR formulation, it is essential to investigate the whole product and its composition, taking into account the specific application. Conducting an investigation into intermediates, such as films, rather than the entire product (artificial leathers) has not been proven to be purposeful.

### 3.3. Variation of Flame Retardant Concentration

The concentration of FR in artificial leathers was varied within the range of 5–30 phr. An exception was ATH, which resulted in a viscosity increase that was too high for coating applications. Therefore, ATH was applied at a maximum concentration of 20 phr. Since the transferability of results from films to artificial leather is not given, only artificial leathers were considered. The characteristics of the burning behavior are summarized in Table 4.

The LOI of all artificial leather samples increased with the addition of more phosphorous FR. For materials with PEster and Phos, no significant improvements above 10 phr were observed. However, org. P and APP showed the highest LOI values at an FR concentration of 30 phr. In general, all phosphorus-containing artificial leathers are slightly sooted during the combustion process, resulting in enhanced total smoke production (TSP). Additionally, all artificial leathers formed a char layer, especially for concentrations above 20 phr. The FR-free reference material ashed without forming a char layer.

The burning rates of the artificial leathers in the horizontal burning test were consistently below the threshold of 100 mm/min. Furthermore, the majority of samples with FRs showed a reduced burning rate in comparison to the FR-free reference material. The change in the Phos concentration did not affect the burning rate significantly. However, org. P and PEster led to a substantial correlation of a decreasing burning rate when increasing the FR concentration. Materials with org. P are inherently self-extinguishing at concentrations exceeding 20 phr. Conversely, an increase in the APP concentration did not result in a decrease in the burning rate.

The characteristic values of cone calorimetry, including TTI, PHRR, and THR, are summarized in Table 4. The ignition of most artificial leathers occurred within 15–20 s after the initiation of the heat supply. Only artificial leathers with high concentrations of Phos and org. P started burning later. Except for PEster, there is no trend regarding decreasing or increasing TTI with increasing FR concentration. The PHRR decreased in all samples compared to the artificial leather without FR. For the samples containing org. P, PEster, and APP, a clear trend of a decreasing PHRR with increasing FR concentration was observed. The THR showed a great variety from which no trend can be derived.

To facilitate comprehension, the trends with increasing FR concentration are summarized in Table 5. A clear trend is not evident. For PEster, the highest LOI was observed with 10 phr, while the best cone calorimetric results and burning rate were achieved at 30 phr. In contrast, for APP, the highest LOI was obtained with 30 phr, yet the lowest burning rate was recorded at 10 phr. The use of Phos or ATH did not yield discernible effects. Concentrations of 5–10 phr led to similar results to those observed in higher concentration samples; although, these concentrations were not adequate for ensuring flame retardancy. It becomes clear once more that the FR has to be selected according to the final application of a material. The composition must be adjusted and tested depending on the requirements of the specific use. In particular, the prediction of fire safety referring to FR concentrations was not possible. Since other properties, such as mechanical stability, must also be taken into account, the lowest possible amount of FR should be used. Irrespective of the concentration, Phos and ATH were not suitable FRs for application in PUR-based artificial leather, at least at the chosen moderate concentrations and with no additional synergist or FR. That may be due to the mode of action, which depends on the oxidation state of phosphorus. The P in Phos has oxidation state I, which is the lowest among all phosphorous FRs examined. It is the only FR that is mainly active in the gas phase, while all the others are charring or intumescence-promoting substances. One potential conclusion is that the combustion of the thin material occurs too rapidly, thereby preventing the initiation of the gas phase reaction. A combination with a delaying FR could provide a remedy.

### 3.4. Combination of Flame Retardants

The application of two synergistically acting FRs can help to suppress the combustion of the material. Therefore, two FRs with distinct mechanisms of action, one charring and the other intumescence-inducing, were combined and incorporated into the intermediate layer of an artificial leather.

**Figure 5:**
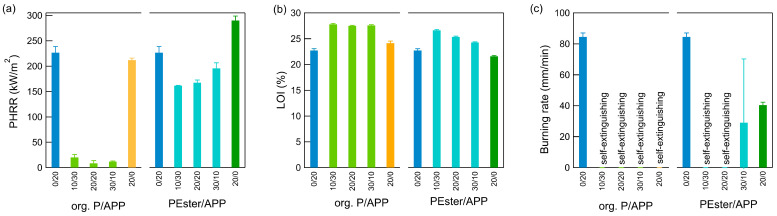
Results of the FR combinations of org. P/APP and PEster/APP in various ratios for (**a**) cone calorimetry (PHRR), (**b**) limiting oxygen index, and (**c**) horizontal burning. (Abbreviation: org. P—organic phosphorus compound; PEster—phosphate ester; APP—ammonium polyphosphate). The total FR concentration was 40 phr.

The combination of APP and org. P, as well as the combination of APP and PEster, were investigated in various ratios, incorporated into artificial leather, and examined through diverse methodologies (Figure 5). Especially, the first combination had a substantial impact, characterized by a significant increase in the LOI and a decrease in the PHRR, reaching almost zero. A similar trend was observed in the combination of APP and PEster, where a decrease in the PHRR was accompanied by an increase in the LOI. In contrast to the APP/org. P combination, the results of the APP/PEster mix depended on FRs’ mixing ratios. The most effective combination was identified as a mixture of 10 phr PEster and 30 phr APP, which was still inferior to all APP/org. P combinations.

In horizontal burning tests, all combinations were self-extinguishing with one exception. Only one PEster/APP sample out of three in the ratio 30/10 phr burned, resulting in a large error bar.

### 3.5. Synergistic Effects

**Figure 6:**
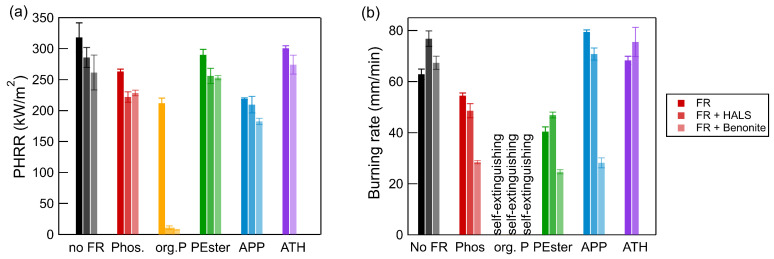
Results of (**a**) cone calorimetry (PHRR) and (**b**) horizontal burning tests of artificial leather with 20 phr FR (darkest color) and 2 phr bentonite or 1 phr HALS (lightest color). (Abbreviation: Phos—phosphinates; org. P—organic phosphorus compound; PEster—phosphate ester; APP—ammonium polyphosphate, ATH—aluminum trihydroxide).

The incorporation of 2 phr bentonite to the formulation of the artificial leather without FR resulted in a slight increase in the LOI value from 20.0% to 20.4%. If 1 phr HALS was used, an increase to 21.4% was observed. The addition of 20 phr of any of the FRs resulted in a discernible enhancement of the fire-retardant properties. The LOI increased slightly (0.3–3.0%) for all composite materials with HALS, while the incorporation of bentonite resulted in a more pronounced rise ranging from 1.2 to 3.8%. Larger impacts could be observed in the cone calorimetry and horizontal burning tests (Figure 6). The incorporation of HALS or bentonite resulted in a decrease in all PHRR values. For artificial leather with org. P, values close to zero could be attained. The heat flux of 25 kW m^−2^ was sufficient to ignite samples; however, the flame extinguished immediately.

In the horizontal burning tests, a pronounced flame-inhibiting effect was observed in samples containing phosphorous FR and bentonite. The burning rate decreased significantly by 51.3 mm/min for APP, 26.1 mm/min for Phos, and 15.7 mm/min for PEster. Another advantageous aspect of the presence of bentonite is the suppression of the afterglow, which was observed for bentonite-free samples.

The findings revealed that the incorporation of both additives into artificial leathers without FR had no substantial impact. On the contrary, the burning rate increased. If phosphorous FR was added, the flammability was reduced, indicating a synergistic effect. It is noteworthy that the incorporation of a single additive did not yield the observed level of fire retardancy, underscoring the necessity for a multifaceted approach to achieve substantial fire safety.

### 3.6. Mechanical Properties and Long-Term Durability

Not only the fire properties, but also the mechanical properties, are decisive for the use of a material as an artificial leather. Therefore, the tensile strength, elongation at break, and permanent folding behavior of the artificial leathers were investigated. The results are summarized in Table S1 of the Supporting Information. Tensile strength and elongation at break were evaluated along both the length and width. The results demonstrated clear differences, which is characteristic when a textile carrier is employed. The artificial leathers showed lower tensile strength in the crosswise direction in general. Comparing all artificial leathers with FRs, the one with ATH showed the lowest values. Artificial leathers with phosphorous-containing FRs showed no significant variation and were similar to the reference artificial leather without FR. In terms of elongation at break, the artificial leathers without FRs and with PEster had the highest values, while the artificial leathers with solid FRs showed significantly lower elongation at break, particularly in the lengthwise direction. In the flex-resistance test, one of five artificial leather samples without FR showed a very fine tear after 100,000 folds. Some of the artificial leathers with phosphorous FR had fine cracks or breaks in the coating as well. Artificial leather with ATH already showed clear cracks after 50,000 folds. In general, the addition of FRs worsened the mechanical properties of artificial leathers but to a reasonable extent.

Selected artificial leathers were characterized by horizontal burning after 4.5 years. Artificial leathers with 20 phr Phos, PEster, ATH, and an artificial leather with 20 phr org. P and 2 phr bentonite were investigated. The results are slightly different after this period but within the same range. The artificial leather with Phos burned with a burning rate of 54.5 ± 1.0 mm/min; after 4.5 years, the rate decreased to 44.2 ± 1.1 mm/min. In contrast, for PEster, an increase in the burning rate was detected (40.4 ± 1.9 → 53.1 ± 1.0 mm/min). The artificial leather with ATH had a higher burning rate compared to the artificial leather without FR, independent from time of testing. The composite material with org. P and bentonite was self-extinguishing immediately after production and after 4.5 years. Although not exactly the same, the values were comparable. Therefore, the durability of the investigated FRs is given within the time frame studied.

### 3.7. Transferability to Other High-Solid PURs

**Figure 7:**
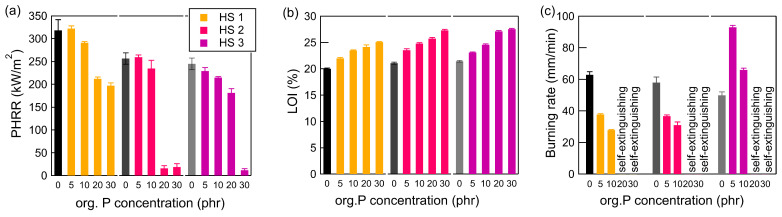
Results of artificial leathers based on different polyurethanes with different methods (**a**) cone calorimetry (PHRR), (**b**) LOI, and (**c**) burning rate. The shades of grey are the artificial leathers without flame retardant (Abbreviation: org. P—organic phosphorus compound; HS—high-solid).

Since the results presented so far were obtained with only one aromatic high-solid prepolymer HS 1, a second aromatic prepolymer HS 2 and an aliphatic prepolymer HS 3 were investigated with org. P, the most effective FR. In general, all PUR materials showed an enhancement in fire retardancy with an increase in the amount of org. P (Figure 7).

Artificial leathers with 5 phr FR exhibited a comparable PHRR to the reference sample without FR. For aromatic HSs (HS 1 and HS 2), significant decreases were obtained for concentrations above 20 phr org. P. The aliphatic PUR (HS 3) showed this decrease with 30 phr org. P. It was observed that at the highest concentration, the ignition of the samples was extremely difficult.

The LOI rose with increasing org. P concentrations, ranging from 5 phr to 30 phr. The increase in LOI for HS 1 was 5.1 percentage points (20.0% → 25.1%); for HS 2, it was 6.2 percentage points (21.1% → 27.3%); and for HS 3, it was 6.1 percentage points (21.5% → 27.6%). Especially, HS 2 and HS 3 are close to being ranked as self-extinguishing [34].

The horizontal burning tests demonstrated that all of the polymers were self-extinguishing with org. P concentrations above 20 phr. The aliphatic polyurethane HS 3 showed an increased burning at FR concentrations of 5 and 10 phr. All other samples displayed an enhanced fire-retardant effect with increasing FR concentrations across all the applied testing methodologies.

## 4. Conclusions

In this study, the flame retardancy of artificial leather samples and films based on high-solid PUR was investigated. Various phosphorous flame retardants were utilized, and the samples were analyzed to determine their effectiveness. Most FRs exhibited a fire-retarding or even inhibitory effect in these thin, flexible materials. However, it was observed that the findings derived from the analysis of one-layer films could not be directly extrapolated to the intermediate layer of artificial leathers that possessed a similar composition.

The application of diverse analytical methods yielded insights into numerous properties, including flammability, heat release, time to ignition, burning rates, and thermal and mechanical stability. Nevertheless, the performance of a given FR in a particular test may not necessarily be indicative of its performance in other tests. This highlights the necessity for a comprehensive and systematic approach to evaluate the performance of FRs in various materials and conditions. Only org. P showed favorable results across all tests. Artificial leathers with org. P showed self-extinguishing burning behavior, a reduced PHRR, and an enhanced LOI. This phenomenon can be attributed to two distinct mechanisms of action that complement each other. In addition to gas phase action, char formation was observed. The results achieved with the other FRs did not indicate the presence of a multistage mechanism. A further improvement could be achieved by combining org. P with APP or bentonite as synergists. For both combinations, a PHRR of almost zero could be reached. All other investigated phosphorous-based FRs showed fire-inhibiting properties as well; however, the optimal FR concentration and combination must be selected based on the intended application of the artificial leather and the requisite burning tests to be passed. The mechanical properties of artificial leather were influenced by the FR. All phosphorous-based-FR-containing artificial leathers showed changes in an acceptable range; only ATH showed a clear deterioration.

The transfer of the findings to multiple high-solid-PUR-based artificial leathers makes the results generally applicable. The results offer deep scientific insight into the working mechanisms of complex multi-component materials but also provide the potential for a fast transfer to the industry since commercially available FRs were used. The knowledge acquired can be applied to enhance the quality of artificial leather products, particularly within the transportation sector, encompassing the automotive and public transport industries, as well as the furniture industry.

Further studies are required to investigate the synergistic effect of bentonite with phosphorous-based FRs. Microstructural analysis with techniques like scanning electron microscopy and infrared spectroscopy will provide insights into structural modifications and chemical interactions. The usage of the combination of org. P and bentonite has the potential to yield consistently good fire retardancy but a decreased amount of phosphorous FR. Given the finite nature of phosphorus and its role as a cost factor in FRs, there is a strategic imperative to minimize the amount of usage.

The utilization of solvent-free polymers, halogen-free flame retardants, and the avoidance of an over-concentration of FR are important milestones in the development of more sustainable materials without compromising performance.

## Figures and Tables

**Table 1:** Properties of all twelve used FRs. FRs used for extensive studies are highlighted in gray.

Flame Retardant	Abbr.	P-Content (%)	N-Content (%)	Mechanism
Phosphinate	Phos	23.3–24	-	Gas phase
Phosphinate		23.5	-	Gas phase
Aluminum-Diethyl-Phosphinate		23.3–24	-	Gas phase
Organic phosphorus compound	org. P	24	-	Charring
Oligomeric phosphate ester	PEster	12.8	-	Charring
Butylated triphenyl phosphate		8.5	-	Charring
Ammonium polyphosphate	APP	26	14	Intumescence
Ammonium polyphosphate		31–32	14–15	Intumescence
Ammonium polyphosphate/Melamine		29–31	15–17	Intumescence
Ammonium polyphosphate/Melamine		30	17	Intumescence
Ammonium polyphosphate/Silane		31.4	14	Intumescence
Aluminum trihydroxide	ATH	-	-	Dilution

**Table 2:** Composition and preparation conditions of different layers for artificial leathers. The intermediate coat used for extensive studies is highlighted in gray.

	High Solid	Abbr.	Crosslinker	Preheating	Gelation Parameter	Scraper Gap
Topcoat	Polycarbonate ester PUR	TC	- ^1^	2 min 90 °C	3 min 160 °C	0.1 mm
Intermediate coat	Aromatic high solid	HS 1	9.2 phr	2 min 80 °C	3 min 165 °C	0.4 mm
Intermediate coat	Aromatic high solid	HS 2	6.9 phr	2 min 80 °C	2 min 160 °C 2 min 170 °C	0.4 mm
Intermediate coat	Aliphatic high solid	HS 3	14.9 phr	2 min 80 °C	4 min 175 °C	0.4 mm
Adhesive Coat	Anionic, aromatic polyether PUR	AC	6.0 ph ^1^	2 min 80 °C	3 min 165 °C	0.1 mm

^1^ Additional processing aids were needed; details are given in the text.

**Table 3:** Schematic summary of results of films and artificial leathers with different FRs, 20 phr each, in comparison to the respective reference without FR (↑ clearly improved fire retardancy, ↗ improved fire retardancy, → no effect, and ↓ clearly worsened fire retardancy).

	Films			Artificial Leather		
	LOI	PHRR	TGA ^1^	LOI	PHRR	TGA ^1^
Phos	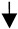	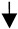	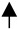	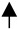	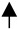	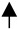
Org. P	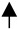	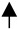	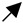	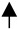	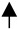	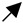
PEster	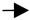	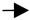	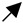	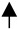	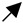	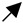
APP	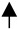	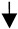	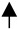	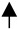	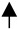	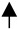
ATH	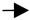	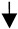	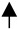	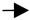	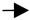	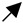

^1^ based on residue masses.

**Table 4:** Comparison of results from artificial leathers with different flame retardants with various concentrations: LOI (DIN EN ISO 4589-2), burning rate (DIN 75200), and cone calorimetry (TTI—time to ignition; PHRR—peak heat release rate; THR—total heat release; TSP—total smoke production; n.d.—not determined). Highlighted in gray are the results of artificial leathers with 20 phr FR.

	FR (phr)	LOI (%)	Burning Rate (mm/min)	TTI (s)	PHRR (kW/m^2^)	THR (MJ/m^2^)	TSP (m^2^)
Without FR	-	20.0	62.9	18.3	318.3	9.1	0.52
Phos	5	21.2	49.1	16.3	249.4	7.4	n.d.
	10	22.3	41.8	16.8	216.1	6.8	n.d.
	20	22.7	54.2	14	260.0	7.3	1.0
	30	22.7	49.3	20.8	230.4	6.2	n.d.
Org. P	5	22.0	37.8	19.7	322.4	10.2	n.d.
	10	23.5	27.9	18.5	291.3	9.8	n.d.
	20	24.2	0	21	212.1	5.1	1.7
	30	25.1	0	24.3	197.1	8.0	n.d.
PEster	5	20.7	53.1	15.5	315.6	9.1	n.d.
	10	21.9	56.1	17.3	311.6	10.2	n.d.
	20	21.6	40.4	17.5	290.1	8.7	1.42
	30	22.0	32.5	19	247.6	7.1	n.d.
APP	5	21.6	60.0	17	276.0	8.8	n.d.
	10	21.7	57.5	23	244.9	5.7	n.d.
	20	22.3	79.5	16	219.0	6.3	0.78
	30	24.4	66.1	16	154.0	5.1	n.d.
ATH	5	19.9	66.7	15	271.7	7.8	n.d.
	10	20.1	57.4	19	270.0	7.8	n.d.
	20	20.3	68.3	19	296.8	9.9	3.8

**Table 5:** Compared and summarized are the testing results of artificial leathers with different FRs giving the trends of efficiency with increasing flame retardant concentration (↑ clearly improved, ↗ improved, → equal, and ↘ worsened).

	LOI	Horizontal Burning	Cone Calorimetry
Phos	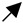	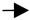	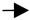
Org. P	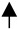	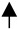	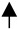
PEster	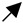	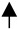	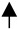
APP	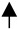	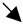	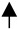
ATH	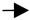	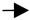	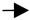

## Data Availability

The original contributions presented in this study are included in this article; further inquiries can be directed to the corresponding author.

## Supplementary Materials

The following supporting information can be downloaded at https://www.mdpi.com/article/10.3390/polym17070841/s1: Table S1: Mechanical properties of artificial leathers based on HS 1 with 20 phr FR: tensile strength and elongation at break (DIN EN ISO 527-3) and permanent folding behavior (DIN EN ISO 32100); Figure S1: Burned films with 30 phr of the corresponding phosphorous flame retardant after cone calorimetry, heat flux 25 kW m^−2^.

## References

[1] Hirschler M.M. (2008). Polyurethane Foam and Fire Safety. Polym. Adv. Technol..

[2] Weil E.D., Levchik S.V. (2004). Commercial Flame Retardancy of Polyurethanes. J. Fire Sci..

[3] Akindoyo J.O., Beg M., Ghazali S., Islam M.R., Jeyaratnam N., Yuvaraj A.R. (2016). Polyurethane Types, Synthesis and Applications—A Review. Rsc Adv..

[4] (2022). Leather-Terminology-Key Definitions for the Leather Trade.

[5] Levchik S.V., Weil E.D. (2004). Thermal Decomposition, Combustion and Fire-Retardancy of Polyurethanes—A Review of the Recent Literature. Polym. Int..

[6] Lu S., Feng Y., Zhang P., Hong W., Chen Y., Fan H., Yu D., Chen X. (2021). Preparation of Flame-Retardant Polyurethane and Its Applications in the Leather Industry. Polymers.

[7] Tabatabaee F., Khorasani M., Ebrahimi M., González A., Irusta L., Sardon H. (2019). Synthesis and Comprehensive Study on Industrially Relevant Flame Retardant Waterborne Polyurethanes Based on Phosphorus Chemistry. Prog. Org. Coat..

[8] Dasari A., Yu Z.-Z., Cai G.-P., Mai Y.-W. (2013). Recent Developments in the Fire Retardancy of Polymeric Materials. Prog. Polym. Sci..

[9] Chattopadhyay D.K., Raju K. (2007). Structural Engineering of Polyurethane Coatings for High Performance Applications. Prog. Polym. Sci..

[10] Shaw S. (2010). Halogenated Flame Retardants: Do the Fire Safety Benefits Justify the Risks?. Rev. Environ. Health.

[11] Kim Y.R., Harden F.A., Toms L.-M.L., Norman R.E. (2014). Health Consequences of Exposure to Brominated Flame Retardants: A Systematic Review. Chemosphere.

[12] Eriksson P., Jakobsson E., Fredriksson A. (2001). Brominated Flame Retardants: A Novel Class of Developmental Neurotoxicants in Our Environment?. Environ. Health Perspect..

[13] Velencoso M.M., Battig A., Markwart J.C., Schartel B., Wurm F.R. (2018). Molekulare Brandbekämpfung—Wie moderne Phosphorchemie zur Lösung der Flammschutzaufgabe beitragen kann. Angew. Chem..

[14] Schartel B. (2010). Phosphorus-Based Flame Retardancy Mechanisms—Old Hat or a Starting Point for Future Development?. Materials.

[15] Braun U., Schartel B. (2008). Flame Retardancy Mechanisms of Aluminium Phosphinate in Combination with Melamine Cyanurate in Glass-Fibre-Reinforced Poly(1,4-Butylene Terephthalate). Macromol. Mater. Eng..

[16] Green J. (1996). Mechanisms for Flame Retardancy and Smoke Suppression—A Review. J. Fire Sci..

[17] Hörold S. (2014). Phosphorus-Based and Intumescent Flame Retardants. Polymer Green Flame Retardants.

[18] Joseph P., Ebdon J.R., Wilkie C.A., Morgan A.B. (2009). Phosphorus-Based Flame Retardants. Fire Retardancy of Polymeric Materials.

[19] Lewin M., Weil E.D. (2001). Mechanisms and Modes of Action in Flame Retardancy of Polymers. Fire Retardant Materials.

[20] Chattopadhyay D.K., Webster D.C. (2009). Thermal Stability and Flame Retardancy of Polyurethanes. Prog. Polym. Sci..

[21] Xue M., Zhang X., Wu Z., Wang H., Gu Z., Bao C., Tian X. (2014). A Commercial Phosphorous-Nitrogen Containing Intumescent Flame Retardant for Thermoplastic Polyurethane. J. Appl. Polym. Sci..

[22] Ma Y., Dang X., Shan Z. (2019). Thermal Analysis and Identification of Potential Fire-Proof Energy Building Material Based on Artificial Leather. J. Therm. Sci..

[23] Zhao J., Duan H., Yang H., Huang Z., Qi D. (2025). Solvent-Free Preparation of Reactive Flame-Retardant Polyurethane Resin for Synthetic Leather Applications. J. Appl. Polym. Sci..

[24] Wang Y., Zhang Y., Liu B., Zhao Q., Qi Y., Wang Y., Sun Z., Liu B., Zhang N., Hu W. (2020). A Novel Phosphorus-Containing Lignin-Based Flame Retardant and Its Application in Polyurethane. Compos. Commun..

[25] Zhang Y.M., Zhao Q., Li L., Yan R., Zhang J., Duan J.C., Liu B.J., Sun Z.Y., Zhang M.Y., Hu W. (2018). Synthesis of a Lignin-Based Phosphorus-Containing Flame Retardant and Its Application in Polyurethane. RSC Adv..

[26] Toldy A., Harakály G., Szolnoki B., Zimonyi E., Marosi G. (2012). Flame Retardancy of Thermoplastics Polyurethanes. Polym. Degrad. Stab..

[27] Ali M.H.M., Rahman H.A., Amirnordin S.H., Khan N.A. (2018). Eco-Friendly Flame-Retardant Additives for Polyurethane Foams: A Short Review. Key Eng. Mater..

[28] Hejna A. (2021). Clays as Inhibitors of Polyurethane Foams’ Flammability. Materials.

[29] Bourbigot S., Duquesne S., Fontaine G., Bellayer S., Turf T., Samyn F. (2008). Characterization and Reaction to Fire of Polymer Nanocomposites with and without Conventional Flame Retardants. Mol. Cryst. Liq. Cryst..

[30] Xie H., Lai X., Zhou R., Li H., Zhang Y., Zeng X., Guo J. (2015). Effect and Mechanism of N-Alkoxy Hindered Amine on the Flame Retardancy, UV Aging Resistance and Thermal Degradation of Intumescent Flame Retardant Polypropylene. Polym. Degrad. Stab..

[31] Lopez-Cuesta J.-M., Jlassi K., Chehimi M.M., Thomas S. (2017). Flame Retardancy Properties of Clay—Polymer Nanocomposites. Clay-Polymer Nanocomposites.

[32] (2015). Reaction-to-Fire Tests—Heat Release, Smoke Production and Mass Loss Rate Part 1: Heat Release Rate (Cone Calorimeter Method) and Smoke Production Rate (Dynamic Measurement).

[33] (2017). Plastics—Determination of Burning Behaviour by Oxygen Index Part 2: Ambient-Temperature Test.

[34] Silva-Santos M.C., Oliveira M.S., Giacomin A.M., Laktim M.C., Baruque-Ramos J. (2017). Flammability on Textile of Business Uniforms: Use of Natural Fibers. Procedia Eng..

[35] (1980). Determination of Burning Behaviour of Interior Materials in Motor Vehicles.

[36] Feng J., Ge Z., Chai C., Wang S., Yu D., Wu G., Luo Y. (2016). Flame Retardant Modification of Waterborne Polyurethane Fabric Coating Agent with High Hydrostatic Pressure Resistance. Prog. Org. Coat..

[37] (2018). Plastics—Determination of Tensile Properties—Part 3: Test Conditions for Films and Sheets.

[38] (2010). Rubber- or Plastic-Coated Fabrics—Physical and Mechanical Tests—Determination of Flex Resistance by the Flexometer Method.

[39] Mequanint K., Sanderson R., Pasch H. (2002). Thermogravimetric Study of Phosphated Polyurethane Ionomers. Polym. Degrad. Stab..

[40] Li H., Ning N., Zhang L., Wang Y., Liang W., Tian M., Chan T.W. (2015). Effect of Content of Organophosphorus on Flame Retardancy Mode of Thermoplastic Polyurethane. Polymer.

[41] Weil E.D., Levchik S.V. (2009). Flame Retardants for Plastics and Textiles: Practical Applications.

[42] Thirumal M., Singha N.K., Khastgir D., Manjunath B.S., Naik Y.P. (2010). Halogen-Free Flame-Retardant Rigid Polyurethane Foams: Effect of Alumina Trihydrate and Triphenylphosphate on the Properties of Polyurethane Foams. J. Appl. Polym. Sci..

[43] Duquesne S., Fontaine G., Cérin-Delaval O., Gardelle B., Tricot G., Bourbigot S. (2013). Study of the Thermal Degradation of an Aluminium Phosphinate–Aluminium Trihydrate Combination. Thermochim. Acta.

[44] Schartel B., Bartholmai M., Knoll U. (2005). Some Comments on the Use of Cone Calorimeter Data. Polym. Degrad. Stab..

[45] Schartel B., Hull T.R. (2007). Development of Fire-Retarded Materials—Interpretation of Cone Calorimeter Data. Fire Mater..

[46] Li H., Ning N., Zhang L., Wang Y., Liang W., Tian M. (2014). Different Flame Retardancy Effects and Mechanisms of Aluminium Phosphinate in PPO, TPU and PP. Polym. Degrad. Stab..

