# Supplementary material for: Phosphorous-Based, Halogen-Free Flame Retardants for Thin, Flexible Polyurethane Artificial Leathers
Source: Polymers (Basel). 2025 Mar 21;17(7):841. doi: 10.3390/polym17070841 (PMC11991176; doi:10.3390/polym17070841)
Supplement: Supplementary file 1 [file polymers-17-00841-s001.zip › polymers-3521910-supplementary.pdf]

Supporting Information

Phosphorous, halogen-free flame retardants for thin, flexible polyurethane artificial leathers

Miriam Bader[1,*], Maren Lehmann[1] and Michael Meyer[1]

[1] FILK Freiberg Institute gGmbH, Meißner Ring 1-5, 09599 Freiberg, Germany
* Correspondence: miriam.bader@filkfreiberg.de; Tel.: +49-3731-366155

Table S1: Mechanical properties of artificial leathers based on HS1 with 20 phr FR: tensile strength and elongation at break (DIN EN ISO 527-3) and permanent folding behavior (DIN EN ISO 32100).

Artificial leather with	Tensile strength N/mm^{-2}		Elongation at break [%]		Permanent folding behavior [grade] 100.000 foldings
	lengthwise	crosswise	lengthwise	crosswise	
Without FR	5.7	4.5	184	194	0/0/1/0/0
Phos	5.9	4.3	68	173	0/0/2/0/2/0
org. P	6.8	4.8	74	166	2/0/2/1/1/3
PEster	6.0	4.5	147	173	0/4/0/0/0/1
APP	6.3	4.5	88	181	0/0/3/1/2/3
ATH	5.2	3.6	75	174	4/5/4/4/5/5

Grade (permanent folding behavior)
0 – no changes
1 – small changes, only detectable with magnifier, white quarry, wrinkling
2 – top layer of coated textile shows fine cracks, only detectable with magnifier
3 – cracks in base layer, only detectable with magnifier
4 – rough cracks in base layer
5 – coating perforated

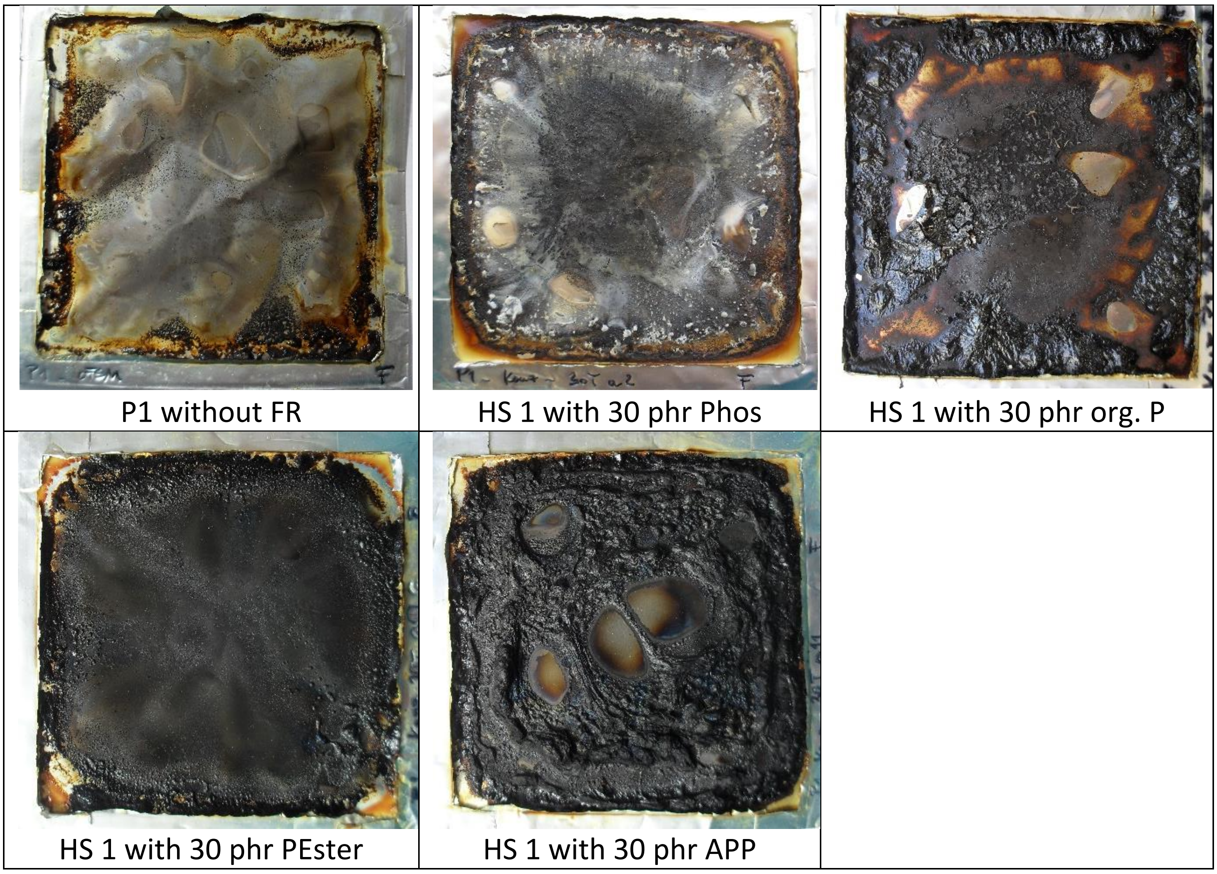

Figure S1: Burned films with 30 phr of the corresponding phosphorous flame retardant after cone calorimetry, heat flux 25 kW m^{-2}.
